# Prevalence of Zika virus neutralizing antibodies in healthy adults in Vietnam during and after the Zika virus epidemic season: a longitudinal population-based survey

**DOI:** 10.1186/s12879-020-05042-2

**Published:** 2020-05-11

**Authors:** Co Thach Nguyen, Meng Ling Moi, Thi Quynh Mai Le, Thi Thu Thuy Nguyen, Thi Bich Hau Vu, Hai Tuan Nguyen, Thi Thu Hang Pham, Thi Hien Thu Le, Le Manh Hung Nguyen, Minh Huong Phu Ly, Chris Fook Sheng Ng, Taichiro Takemura, Kouichi Morita, Futoshi Hasebe

**Affiliations:** 1 Graduate School of Biomedical Sciences, Nagasaki University, Nagasaki, Japan; 2 Department of Virology, Institute of Tropical Medicine, Nagasaki University, Sakamoto 1-12-4, Nagasaki, 852-8523 Japan; 3 Program for Nurturing Global Leaders in Tropical and Emerging Communicable Diseases, Nagasaki University, Nagasaki, Japan; 4 National Institute of Hygiene and Epidemiology, Hanoi, Vietnam; 5 Tay Nguyen Institute of Hygiene and Epidemiology, Tay Nguyen, Dak Lak Vietnam; 6 School of Tropical Medicine and Global Health, Nagasaki University, Nagasaki, Japan; 7 Vietnam Research Station, Center for Infectious Disease Research in Asia and Africa, Institute of Tropical Medicine, Nagasaki University, Nagasaki, Japan

**Keywords:** Zika virus, Zika virus infection, Seroprevalence, Seroepidemiologic studies, Neutralizing antibodies, Vietnam

## Abstract

**Background:**

Between 2016 and 2019, 265 cases of Zika virus (ZIKV) infection were reported in Vietnam, predominantly in southern Vietnam. In 2016, a case of ZIKV-associated microcephaly was confirmed in the Central Highlands, and several members of the infant’s family were confirmed to be infected with ZIKV. The study aims to determine the level of immunity to ZIKV in the general population of the ZIKV epidemic region.

**Methods:**

A total of 879 serum samples were collected from 801 participants between January 2017 and July 2018, during and after the ZIKV epidemic in Vietnam. The samples were tested for anti-ZIKV immunoglobulin M (IgM) and immunoglobulin G (IgG), and anti-dengue virus (DENV) IgG antibodies using enzyme-linked immunosorbent assays (ELISA). Plaque-reduction neutralization test (PRNT) for ZIKV was performed on all samples, and for DENV on the samples that ZIKV neutralizing antibody positive.

**Results:**

A total of 83 (10.3%) participants had anti-ZIKV IgM. Of the 83, 6 were confirmed to be ZIKV antibodies positive using PRNT and anti-ZIKV IgG ELISA. Of the 718 participants who were anti-ZIKV IgM negative, a further 3 cases were confirmed as positive for antibodies against ZIKV. Of the 9 participants with ZIKV infection, 5 lived in the same village as the infant with ZIKV-associated microcephaly and the other 4 lived in 2 neighboring communes. Repeat samples were collected from the 83 ZIKV IgM positive participants 1.5 years after the first collection. No new cases of ZIKV infection were detected. In addition, 2 of 3 participants with anti-ZIKV NS1 IgG demonstrated a 4- to 8-fold increase in ZIKV neutralizing antibody titer.

**Conclusions:**

ZIKV was present in the area around Krong Buk, with the rate of ZIKV-specific antibodies was 1.1% in the community since at least 2016. While the low levels of circulation together with low seroprevalence suggests a limited outbreak in the region, the results also reflect on low levels of protective immunity to Zika within the population. These results provide a better understanding of the current ZIKV epidemic status in the region and demonstrate a need for implementation of more effective ZIKV infection control measures.

## Background

Zika virus (ZIKV) was first isolated from Rhesus monkeys in the Zika forest in Uganda in 1947 [1]. It is a mosquito-borne virus belonging to the genus *Flavivirus* and family *Flaviviridae* [2], which is spread from person to person mainly through the bite of infected *Aedes aegepti* and *Aedes albopictus* mosquitoes [3]*.* ZIKV can also be transmitted through sexual intercourse or body fluids [4]. Common symptoms are rash, fever, arthralgia, and conjunctivitis [5]. While ZIKV infection is sometimes associated with only mild symptoms, it can also lead to severe complications including Guillain-Barré syndrome [6] and microcephaly in infants [7].

The first reported ZIKV epidemic occurred in Yap Island, Federated States of Micronesia, in 2007, with an estimated 5000 of a total of 6800 residents infected [5]. The second reported epidemic occurred in French Polynesia in 2013 and 2014, with an estimated 28,000 people infected, comprising approximately 11% of the population [8, 9]. As many as 1.3 million people may have been infected in an epidemic in 14 states of Brazil in 2015 and 2016 [10]. During the epidemic period, there was an exponential increase in the number of cases of infants born with microcephaly suspected to be associated with ZIKV [7, 10]. According to a July 2019 WHO report there has been evidence of ZIKV transmission in 87 countries and territories in the Americas, Africa, Southeast Asia, and the Western Pacific region [11].

While Southeast Asia has been known as a ZIKV endemic region for more than 60 years, large ZIKV epidemics has only been reported recently [12]. Although the virus has been first isolated from mosquitoes in Malaysia in 1966, the first human cases were only reported in 1977 [13]. In 2016, a total of 455 cases were confirmed in Singapore [14] and, in Thailand, 386 cases were reported in 29 out of 76 provinces from 2015 to 2017 [15]. During this period, cases of ZIKV infection were also reported in other Southeast Asia nations including Malaysia [16] and Myanmar [17]. In 2016, 3 tourists were confirmed to have ZIKV infection after visiting Vietnam [18–20]. As of June 2019, a total of 265 cases has been reported in Vietnam, most of which occurred in Ho Chi Minh City [21–24]. Additionally, in 2016, a case of Zika-associated microcephaly was reported in the Central Highlands of Vietnam and 5 family members and 2 neighbors were confirmed positive for ZIKV infection [25]. Despite the endemicity for dengue and the high density of mosquito vectors, the numbers of cases of ZIKV infection in Vietnam remain substantially lower than the number of cases of dengue. Vietnam lies within the tropical zone where *Aedes aegypti* mosquitoes are endemic. While neighboring areas have reported ZIKV outbreaks in recent years, there are limited data available on the extent of ZIKV infection in local populations in Vietnam. Additionally, it has been hypothesized that dengue hyperendemicity may lead to cross-reactive immunity toward ZIKV, thus limiting the size of ZIKV epidemics in Southeast Asia. However, there were limited seroprevalence data to support this hypothesis. Cross-reactivity between ZIKV and DENV antibodies has led to difficulties in the interpretation in some studies [26]. Annually, Vietnam reports approximately 100,000 dengue cases. The incidence of dengue has remained stable over the past few decades in Vietnam [27], and dengue seroprevalence remains high, with up to 64% of the adult population being seropositive [28]. Recent studies have suggested that while DENV is cross-reactive with ZIKV, the level of cross-neutralization and hence disease protection is limited [29–31]. In Vietnam, the number of ZIKV infections peaked at 219 in 2016, and has subsequently decreased, with only one reported case in 2019 [32]. The purpose of this study was to determine the seroprevalence of ZIKV antibodies among the population in Vietnam during and after the 2016 Zika epidemic using ZIKV and DENV neutralizing assays to elucidate the extent of the ZIKV epidemic in the local population.

## Methods

### Ethics approval and consent to participate

Ethics approval for this study was obtained from the ethics committee of the National Institute of Hygiene and Epidemiology, Ministry of Health, Vietnam (IRB-VN01057–45/2016) and the Ethics Committee of the Institute of Tropical Medicine, Nagasaki University, Japan (08061924–7).

### Sample collection

The samples were collected from participants selected using simple random sampling by means of a lottery method in Krong Buk District, Dak Lak Province where a case of ZIKV-associated microcephaly had been reported [25]. The population density in this region is 181 people/km^2^ with the estimated total population of 65,000 living in an area of 357.82 km^2^. In January 2017, 3 months after the case of microcephaly was reported, blood samples were collected from healthy adults in the community with places of residence distributed across all communes in Krong Buk District. None of the study participants were hospitalized for an acute illness during the study period. In this study, we estimated the proportion of persons with ZIKV infection in Krong Buk District.

The required sample size was calculated assuming a precision/absolute error (d) of 4% and a proportion with a 95% level of confidence (Z_1-α/2_ = 1.96) [33]. Samples were processed within 24 h of collection and stored at − 80 °C prior to testing.

### In-house Zika virus immunoglobulin M enzyme-linked immunosorbent assay

The samples were screened for ZIKV IgM using an in-house ZIKV IgM ELISA kit. This method was adapted from Dengue Virus IgM Capture DxSelect (Focus Diagnostics, Cypress, CA, USA) [34] by replacing DENV antigen with ZIKV antigen. The modified in-house ZIKV IgM ELISA has been utilized in other studies [25, 32, 35, 36]. Samples were first diluted 1:100 using sample diluent solution. The 96-well IgM Capture plate (Dengue Virus IgM Capture DxSelect, Focus Diagnostics) were soaked with 1X wash buffer solution for 5 min, and wells were decanted. A total of 100 μL of diluted serum sample was added and incubated at 37 °C for 1 h. The plates were then washed 3 times with 1X wash buffer solution. Next, 100 μL of ZIKV antigen (MR-766, 10^5^ PFU/mL) was added and incubated at room temperature (RT) for 1 h. The wells were then washed with 1X wash buffer solution for a total of 3 times to remove excess antigen. Next, 100 μL of affinity-purified and peroxidase-conjugated mouse anti-flavivirus antibodies was added to all wells and the plate was incubated at RT for 30 min. The plates were washed 3 times to remove residual conjugate. Next, 100 μL of tetramethylbenzidine (TMB) substrate solution and horseradish peroxide was added to each well and the plate was then incubated at RT for 10 mins in the dark. A total of 100 μL of stop solution (1 M sulfuric acid) was added to each well to stop the reaction. Finally, the plates were read at 450 nm of the optical density (OD) using an ELISA plate reader (Multiscan ELISA reader, Thermolab System, Tokyo, Japan). OD values which were ≥ 2 times that of the negative control (N) is regarded as positive (P).

### Detection of dengue virus immunoglobulins M and G (IgM and IgG), and Zika virus immunoglobulin-G by enzyme-linked immunosorbent assay (ELISA)

In addition to the detection of anti-ZIKV IgM antibodies by ELISA, anti-dengue IgM antibodies (Vircell, Granada, Spain) and anti-dengue IgG antibodies (Vircell) were determined according to manufacturer’s instructions. Human Anti-Zika Virus IgG ELISA Kit (R&D Systems, Inc. Minneapolis, MN, USA) was used to test samples for ZIKV-specific NS1-antigen-reactive IgG antibodies in the samples that exhibited anti-ZIKV IgM antibodies and neutralizing antibodies to ZIKV according to manufacturer’s instructions. OD values which were ≥ 2 times that of the negative control (N) were regarded as positive (P).

### Plaque-reduction neutralization test

Serum samples were screened for the presence of neutralizing antibodies to ZIKV using a plaque-reduction neutralization test (PRNT). Serum samples were inactivated at 56 °C for 30 min before testing. In the first PRNT screening for ZIKV, the serum was diluted 10 times in the EMEM (Nissui Pharmaceutical Co., Ltd., Tokyo, Japan) containing 2% FBS, then the serum samples were serially diluted 2-fold (1:10–1:10240). The PRNT was performed in replicates of 2 for ZIKV (MR766 laboratory strain) and all 4 DENV serotypes (DENV-1 01–44 strain; DENV-2 TLC-30 strain; DENV-3 CH53469 strain; DENV-4 SLMC318 strain). At each dilution, 50 μL of serum sample was mixed with 50 μL virus containing 100–200 plaque forming units (2000–4000 PFU/mL). The immune virus-complex mixture was then incubated at 37 °C for 1 h. A total of 50 μL of virus-immune complex mixture was then added onto BHK cell monolayers in 12 well plates (Corning Costar, Sigma-Aldrich, St. Louis, Missouri, USA) and incubated at 37 °C in 5% CO_2_ for 1 h. After incubation, overlay medium (2 mL of EMEM/ 1% methylcellulose (Wako Pure Chemical Industries Ltd., Osaka, Japan), in 2% FBS) was added into each well. The plates were incubated at 37 °C in 5% CO_2_ for 4–6 days until visible plaque formation. Cells were then fixed using 4% paraformaldehyde phosphate buffer solution (Wako) for 1 h at RT and then stained with 1.25% crystal violet (Wako). The plaques were then counted by naked eye. The neutralization titers, PRNT_50_ and PRNT_90_, were defined as the highest serum dilution which reduced the number of plaques by 50 and 90% respectively. The presence of neutralizing antibodies was defined as ZIKV PRNT_50_ titer ≥20. The criteria to confirm the case exposed to ZIKV that has neutralizing antibodies to ZIKV with PRNT_50_ titer ≥20, and 4 fold higher compare to DENV titers. The interpretation of probable ZIKV infection was according to WHO guidelines for laboratory testing for Zika virus infection in which samples that were positive for ZIKV IgM and negative for DENV IgM was interpreted as ZIKV probable case [37].

### Data analysis

Descriptive analyses were performed, using frequencies and percentages for categorical variables; and means and standard deviations (SDs) for continuous variables. Odds ratios (ORs) and the 95% confidence intervals (CIs) were used to estimate the relative likelihood of ZIKV infection in each group. Multiple logistic regression was used to assess the association of sex, ethnicity, and age group with ZIKV infection. The analyses were performed using Stata 14.1 (StataCorp LP, College Station, Texas, USA) with 5% level of significance and two-tailed *p* values were reported. The map was created using QGIS software 3.8.2.

## Results

### Demographic characteristics of participants

The serological surveillance study participants were randomly selected from the community of Krong Buk District. Serum samples were collected from 801 of 65,000 residents (1.2%) in January 2017. The median age is 33 years, dominated by those in the age group between 16 and 60 years (*n* = 663 or 82.7%). A total of 87 samples were collected from children under the age of 15 years (10.9%). The proportion of females in the study was more than 73%. In the analysis by ethnicity, 503 samples (62.8%) were from the Kinh group, which is the major ethnic group in Vietnam, while a minority ethnic group Ede made up 290 samples (36.2%), followed by 8 participants of other minor origin (1%). The number of pregnant women who participated in this study was 66 (8.24%).

### Zika virus and dengue seroprevalence

**Fig. 1:**
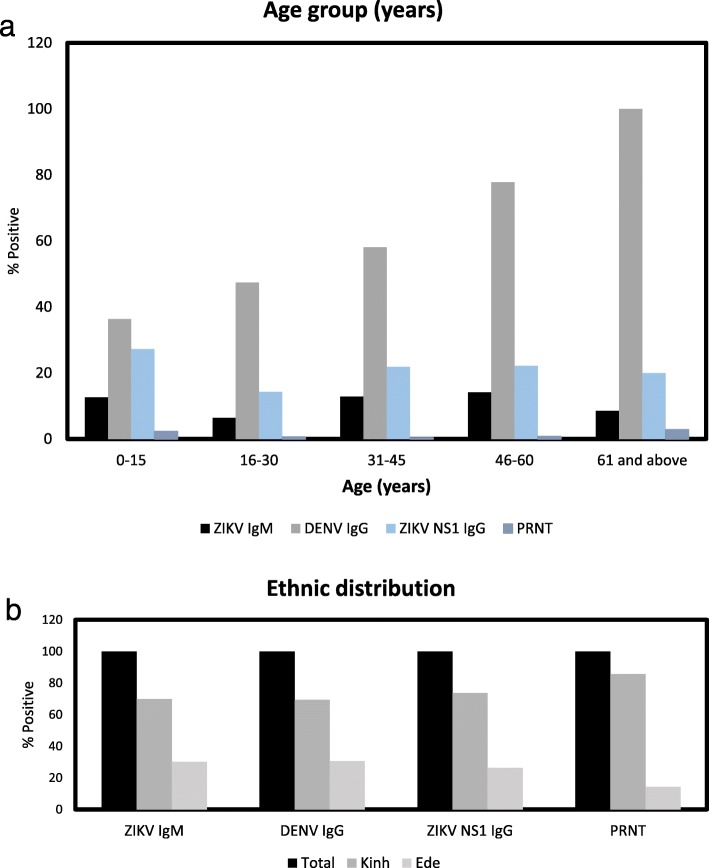
Seroprevalence of Zika virus (ZIKV) antibodies in Central Vietnam, 2017–2018. The seroprevalence of Zika virus antibodies according to (**a**) age and (**b**) ethnicity. A total of 83 participants (83/801, 10.3%) demonstrated anti-ZIKV IgM antibodies as determined by using enzyme-linked immunosorbent assays. ZIKV neutralizing antibodies was determined by using plaque-reduction neutralization test (PRNT) and for confirming infection to ZIKV. Prevalence rates (anti-DENV IgG antibodies and anti-ZIKV NS1 IgG antibodies) was determined by using a total of 83 samples and 88 samples, respectively

Of the 801 samples tested, 83 (10.3%) were positive for anti-ZIKV IgM antibodies (Table 1) with an average P/N ratio of 3.30 ± 1.48. While the P/N ratio is not a quantitative method, the ratio was used as a means to determine antibody levels of 83 samples, for the presence or absence of anti-ZIKV IgM antibodies. All participants who tested positive were asymptomatic at the time of sample collection. The prevalence of anti-ZIKV IgM antibodies varied moderately by age (*p* = 0.05) and was highest in the 46–60 year age group (14.2%) (Fig. 1(a)). The seroprevalence of ZIKV IgM did not differ significantly according to sex or ethnicity. Only one of the 66 pregnant women (1.5%) was positive for anti-ZIKV IgM antibodies.

**Table 1:** Demographic characteristics of the study participants (*N* = 801)

Variables		n	ZIKV IgM positive (%)	Crude OR (95% CI)	*P*-value	DENV IgG positive (%) (*n* = 83)	Anti-ZIKV NS1 IgG positive (%) (*n* = 88)	PRNT_50_ positive to ZIKV (%)
**Age group (years)**	≤15	87	11 (12.6)	1.00 (Reference)	0.05	4/11 (36.3)	3/11 (27.3)	2/87 (2.3)
	16–30	295	19 (6.4)	0.48 (0.22–1.04)		9/19 (47.4)	3/21 (14.3)	3/295 (1.0)
	31–45	241	31 (12.8)	1.02 (0.49–2.13)		18/31 (58.1)	7/32 (21.9)	3/241 (1.2)
	46–60	127	18 (14.2)	1.14 (0.51–2.55)		14/18 (77.8)	4/18 (22.2)	1/127 (0.8)
	≥60	35	3 (8.5)	0.65 (0.17–2.48)		3/3 (100)	1/5 (20)	3/35 (8.6)
	NA^a^	16	1 (6.3)			1/1 (100)	1/1 (100)	1/16 (6.3)
**Sex**	Female	590	59 (10)	1.00 (Reference)	0.57	32/59 (54.2)	10/64 (15.6)	7/590 (1.2)
	Male	211	24 (11.4)	1.15 (0.7–1.91)		17/24 (70.8)	9/24 (37.5)	6/211 (2.8)
**Ethnicity**	Ede	290	25 (8.6)	1.00 (Reference)	0.16	15/25 (60.0)	5/26 (19.2)	3/290 (1.0)
	Kinh	503	58 (11.5)	1.42 (0.87–2.33)		34/58 (58.6)	14/62(22.6)	10/503 (2.0)
	Other^b^	8	0					0/8 (0.0)
**Pregnancy**		66	1 (1.5)			0/1(0.0)	0/1(0.0)	0/66 (0.0)

*CI* confidence interval, *DENV* dengue virus, *IgG* immunoglobulin G, *IgM* immunoglobulin M, *NA* not available, *OR* odds ratio, *ZIKV* Zika virus, ssIKV NS1 IgG positive, ZIKV antibodies were determined using anti-ZIKV NS1 IgG ELISA (P/N ratio ≥ 2); PRNT_50_ positive to ZIKV was defined as plaque-reduction neutralization test titers with a ≥ 50% reduction in ZIKV plaque-forming units at a serum dilution of ≥1:20

^a^ Participants with missing information on age were excluded from the multivariate logistic regression analysis

^b^ Participants whose ethnicity was categorized as “Other” were combined with the “Ede” ethnic group in the multivariate logistic regression analysis

All 83 participants with ZIKV anti-IgM antibodies were negative for dengue IgM on ELISA; however, 49 (59.0%) ZIKV IgM positive cases were detected as positive for Dengue IgG ELISA (Table 1). DENV IgM and DENV IgG antibodies was determined to examine possible cross-reactivity to ZIKV infection. The distribution of the ZIKV IgM antibodies positive cases, as well as DENV IgG antibodies positive cases, was observed in all age groups in this study (Fig. 1a). All testing results showed higher positive rates in the majority Kinh ethnic group than those in the minority Ede ethnic group (Fig. 1b).

### Neutralizing antibody levels to Zika virus and dengue virus serotypes 1–4

All 801 samples were first screened to detect the presence of neutralizing antibodies to ZIKV. Of the 83 ZIKV IgM-positive samples, 8 demonstrated neutralizing antibodies to ZIKV (PRNT_50_ = 1: 40 to 1: 640). Of the 8 samples, 3 (Z141a, Z153a, Z735a) exhibited ZIKV antibody titers that were > 4-fold higher than the antibody titers for all 4 DENV serotypes (PRNT_50_ ZIKV = 1: 160 to 1: 640) (Table 2). The remaining 5 samples (Z120a, Z138a, Z140a, Z420a, Z476a) were classified as probable ZIKV infections (PRNT_50_ ZIKV = 1: 40–1: 640).

**Table 2:** Anti-Zika virus immunoglobulin M and G levels of 13 participants with neutralizing antibodies to Zika virus epidemic) (*N* = 879)

	ELISA results			Neutralizing antibody titer (PRNT_50_)					Neutralizing antibody titer (PRNT_90_)				
Sample ID	ZIKV IgM(P/N ratio)	DENV IgM(Index Value)	Anti-ZIKV NS1 IgG (P/N ratio)	ZIKV	DENV-1	DENV-2	DENV-3	DENV-4	ZIKV	DENV-1	DENV-2	DENV-3	DENV-4
**During epidemic (January 2017)**													
**Z120a**^**a**^	**3.3**	0.1	**2.2**	160	320	320	< 20	< 20	< 20	320	80	< 20	< 20
**Z140a**	**2.3**	0.3	**4.7**^**a**^	80	40	320	< 20	< 20	< 20	< 20	40	< 20	< 20
**Z141a**	**4.9**	0.1	**3.4**	**640**	160	80	< 20	< 20	**40**	80	< 20	< 20	< 20
**Z153a**^**b**^	**2.8**	0.3	**3.2**	**160**	< 20	< 20	< 20	< 20	**40**	< 20	< 20	< 20	< 20
**Z476a**	**4.7**	0.5	**43.9**^**a**^	640	320	1280	20	< 20	**80**	320	160	< 20	< 20
**Z735a**	**2.8**	0.6	**8.1**	**320**	< 80	< 80	< 80	< 80	**80**	< 80	< 80	< 80	< 80
**Z147a**^**a**^	1.6	ND	**4.5**	320	80	160	20	40	**80**	< 20	80	< 20	< 20
**Z472a**	1.5	ND	**46.1**	**5120**	160	160	80	40	**640**	80	40	80	< 20
**Z606a**	1.6	ND	**10.6**	**1280**	< 20	< 20	< 20	< 20	**160**	< 20	< 20	< 20	< 20
Z138a	**4.6**	0.6	0.9	40	320	80	< 20	< 20	< 20	160	< 20	< 20	< 20
Z420a^b^	**3.7**	0.1	0.2	160	160	160	20	< 20	**20**	20	40	< 20	< 20
Z587a	1.2	ND	0.2	320	640	640	1280	160	**80**	320	320	640	20
Z591a	1.2	ND	0.6	640	640	640	1280	80	**80**	320	160	640	< 20
**Post-epidemic (July 2018)**													
**Z120b**^**a**^	ND	ND	**2.3**	80	640	160	1280	20	**20**	320	40	320	< 20
**Z140b**	ND	ND	**5.2**	**640**	< 20	160	< 20	20	**20**	< 20	80	< 20	< 20
**Z141b**	ND	ND	**2.7**	**640**	160	20	160	< 20	**80**	80	< 20	40	< 20
**Z476b**	ND	ND	**4.7**	**2560**	320	320	320	80	**160**	80	80	160	< 20
**Z735b**	ND	ND	**3.5**	80	320	80	320	80	< 20	320	20	160	20
Z138b	ND	ND	0.5	80	40	< 20	40	< 20	< 20	20	< 20	20	< 20

*DENV* dengue virus, *DENV1–4* dengue virus serotypes 1–4, *ELISA* enzyme-linked immunosorbent assay, *IgG* immunoglobulin G, *IgM* immunoglobulin M, *N* negative, *ND* not detected, *P* positive, *PRNT*_*50*_ plaque-reduction neutralization test with neutralization defined as ≥50% reduction in challenge virus plaque-forming units, *PRNT*_*90*_ plaque-reduction neutralization test with neutralization defined as ≥90% reduction in challenge virus plaque-forming units, *ZIKV* Zika virus

Figures in bold indicate positive results_._^a^ ZIKV antibodies were determined using anti-ZIKV NS1 IgG ELISA (P/N ratio ≥ 2). ^b^ Z153 and Z420 were not available during the post endemic collection

Consecutive samples were collected from 78 of the 83 participants with anti-ZIKV IgM antibodies collected from the same participants 18 months after the first sample collection. Second consecutive samples were not collected from 5 participants who was not available at the time of second sample collection, including two (Z153 and Z420) of the 8 participants with ZIKV neutralizing antibodies in their initial sample. Two participants (Z120 and Z735) experienced a 2- to 4-fold decrease, and 2 participants (Z140 and Z476) experienced a 4- to 8-fold increase in their ZIKV neutralizing antibody titers (Table 2). Of the 83 participants with anti-ZIKV IgM antibodies, 5 (6.0%; Z140, Z141, Z153, Z476 and Z735) had ZIKV neutralizing antibody titers that were at least 4-fold greater than their antibody titers against the 4 DENV serotypes tested.

Of the 718 samples that were negative for anti-ZIKV IgM antibodies, 5 (Z147a, Z472a, Z606a, Z587a and Z591a) demonstrated neutralizing antibodies to ZIKV (PRNT_50_ = 1:320–1:5120). Among the 5 samples, 2 samples (Z472a and Z606a) demonstrated a 4-fold or greater level of neutralizing antibodies to all 4 DENV serotypes (Table 2). In addition, by testing all 879 samples collected in both rounds using PRNT, 13 participants (1.6%, *N* = 801) had detectable ZIKV neutralizing antibodies. Overall, 7 participants (0.9%, Z140, Z141, Z153, Z476, Z735, Z472 and Z606) demonstrated ZIKV neutralizing antibody titers that were at least 4-fold higher than their antibody titers to all 4 DENV serotypes, whereas the other 6 participants (0.8%) demonstrated comparable levels of ZIKV and DENV neutralizing antibodies. Thus, the results suggest that the 7 participants had been exposed to ZIKV during the 2016 ZIKV epidemic.

### Anti-Zika virus NS1 immunoglobulin G levels in cases of probable Zika virus infection

Levels of anti-ZIKV NS1 IgG antibodies in the 83 samples that exhibited anti-ZIKV IgM antibodies and five samples that demonstrated ZIKV neutralizing antibodies but negative for anti-ZIKV IgM antibodies (*N* = 88) were determined using anti-Zika Virus NS1 IgG ELISA (R&D Systems). Anti-ZIKV NS1 IgG assays are useful for confirming ZIKV infection because they are highly specific and possess minimal cross-reactivity to other flaviviruses [38]. Sixteen out of 83 ZIKV IgM positive samples (19.3%) demonstrated ZIKV NS1 specific IgG antibodies, with a mean of P/N ratio of 5.7 ± 10.3 (data not shown). Among 8 of 83 ZIKV IgM positive samples that demonstrated neutralizing antibodies to ZIKV, 6 samples (Z120a, Z140a, Z141a, Z153a, Z476a and Z735a) were also positive for anti-ZIKV NS1 IgG by using ELISA. All of 3 samples (Z141a, Z153a and Z735a) that demonstrated a 4-fold or greater ratio of ZIKV neutralizing antibody titers to DENV antibody titers also demonstrated ZIKV NS1 IgG antibodies (P/N ratio = 3.1–8.1). These results confirm that these 3 participants were exposed to ZIKV infection. Three of the 5 samples (Z120a, Z138a, Z140a, Z420a, Z476a) with ZIKV neutralizing antibodies were also confirmed positive for anti-ZIKV NS1 antibodies. The Anti-ZIKV NS1 IgG levels in the second samples collected in July 2018 were comparable to the levels in the first samples.

Three of 5 samples (Z147, Z472, Z587, Z591 and Z606) that demonstrated ZIKV neutralizing antibodies (*N* = 718, ZIKV IgM negative samples) were also positive for anti-ZIKV NS1 IgG antibodies. Two samples (Z472 and Z606) were positive for both ZIKV neutralizing antibodies and ZIKV NS1 IgG (P/N ratio = 10.6 and 46.1 respectively). In addition, one sample (Z147) was also positive for anti-NS1 antibodies with a P/N ratio of 4.5. Finally, a total of 19 samples demonstrated anti-ZIKV NS1 IgG antibodies, including 16 of 83 samples that exhibited anti-ZIKV IgM antibodies positive and three samples that showed ZIKV neutralizing antibodies but negative for anti-ZIKV IgM antibodies (Table 1). However, out of the 19 samples that were positive for anti-ZIKV NS1 IgG antibodies, only 9 (Z120, Z140, Z141, Z153, Z476, Z735, Z147, Z472 and Z606) exhibited neutralizing antibodies against ZIKV with PRNT_50_ titers ≥1:20.

**Fig. 2:**
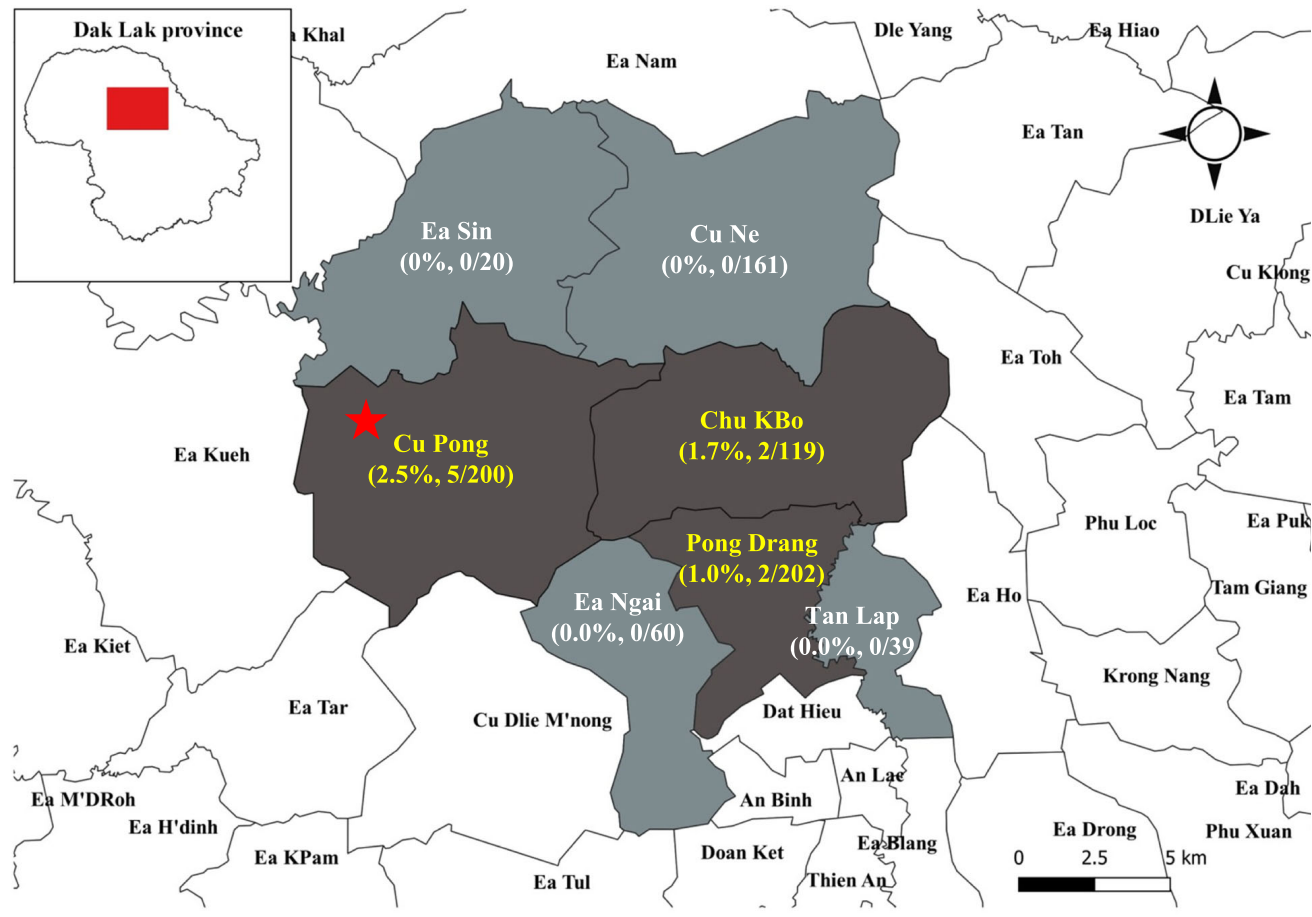
Zika virus (ZIKV) antibody seroprevalence in the survey districts, Dak Lak Province, Central Vietnam. Zika virus seroprevalence was determined by testing serum samples of residents for ZIKV anti-NS1 IgG antibodies by using an enzyme-linked immunosorbent assay (ELISA), and plaque-reduction neutralization test (PRNT). The dark gray shading indicates villages (local administrative units) in which anti-ZIKV antibodies were found in one or more residents. The light gray shading indicates villages in which all study participants tested negative for ZIKV antibodies. N indicates the number of residents who were tested for ZIKV antibodies, and percentage of confirmed cases were indicated in brackets. Star (★) indicates Cu Pong commune, in which a ZIKV-associated microencephaly case has been detected in 2016. Image was created by using QGIS 3.8.2 and shapefile was obtained from https://gadm.org/. The 2020 GADM license allows data re-use for academic and other non-commercial purposes (https://gadm.org/license.html, last accessed: 16th April 2020)

In summary, out of 801 participants tested in this study, by using two methods: the anti-NS1 IgG ELISA and PRNT, we determined that 9 participants (1.12%, Z120, Z140, Z141, Z147, Z153, Z735, Z476, Z472 and Z606) had ZIKV infection. Of these 9 participants, 5 (Z120, Z140, Z141, Z147 and Z153) lived in Cu Pong commune. An infant with microencephaly and her immediate family members has been confirmed confirmed positive for ZIKV infection in the Cu Pong commune in 2016 [25]. This study however excludes samples from our previous study [25]. The 4 remaining cases (Z472, Z476, Z606 and Z735) were found in 2 neighboring communes, two cases in Chu Kbo (1.7%, Z472 and Z476) and two other cases in Pong Drang village (0.99%) (Fig. 2).

## Discussion

In this study, we determined the anti-ZIKV IgM and IgG antibodies among 801 participants who were recruited during ZIKV epidemic in Vietnam. Of the 83 first samples that demonstrated ZIKV IgM antibodies, only 8 (1.0%) had ZIKV neutralizing antibodies (PRNT = 1:40–1:640) with 3 cases exposed to ZIKV infection confirmed using PRNT_50_ with ZIKV neutralizing titer ≥4fold of DENVs neutralizing titer. While anti-IgM ZIKV antibodies may have lower specificity than other laboratory assays, the test is useful for identifying probable ZIKV cases during an outbreak [32, 39]. Due to relatively short detection window for ZIKV IgM antibodies, a combination of tests that determine ZIKV neutralizing antibodies and ZIKV-specific IgG antibodies would be useful for long-term seroprevelance studies. As such, ZIKV IgM test was used to determine ZIKV IgM antibodies during the ZIKV outbreak in Vietnam in 2016, and ZIKV-specific IgG ELISA and PRNT was included in this study. In further tests by using the anti-ZIKV NS1 IgG test, 3 of the 5 probable cases were confirmed positive for anti-ZIKV NS1 IgG antibodies. By using both PRNT (ZIKV, DENV1–4) and anti-ZIKV NS1 IgG assays in all 83 participants that demonstrated cross-reactive ZIKV IgM antibodies, only 6 (7.2%) samples fulfilled the criteria of ZIKV PRNT ratio > 4 and presence of ZIKV-specific NS1 cross-reactive antibodies, confirming that these 6 individuals had recently become infected with ZIKV. Using samples from the same participants, the levels of antibodies were determined 18 months after the first sample collection (post-ZIKV epidemic). Among these 6 confirmed cases, 3 samples that were only ZIKV NS1 IgG antibodies positive during the first collection also demonstrated high ZIKV neutralization titers 18 months later. None of the ZIKV seropositive participants had an international travel history, suggesting that local transmission in the area.

While the prevalence of anti-ZIKV IgM antibodies was highest in the 46–60 year age group, there were no significant discrepancy in anti-ZIKV IgM seropositivity rates across age groups, indicating that the risk of ZIKV infection is homologous across different age groups. In contrast, a high proportion of participants demonstrated DENV IgG antibodies, with seropositivity increasing with age [37, 40, 41]. These results are consistent with those of other studies, indicating association with longer exposure due to persistent DENV endemicity. While there were high levels of DENV seropositivity (49/83; 59.0%), indicating DENV exposure in the community, the overall seropositive rates for ZIKV remains low (9/801; 1.1%). While low ZIKV seroprevalence, a proxy of protection, indicates vulnerability of the population of the region to the ZIKV epidemic, the results also suggest that DENV may offer limited cross-protection against ZIKV.

Among the participants, there was no significant difference in ZIKV IgM seroprevalence according to sex. The higher percentage of female participants in this study is due to socioeconomic factors in this region, as adult males have a higher tendency to travel for employment opportunities in urban areas. While statistically insignificant, the proportion of ZIKV IgM seropositivity in the Kinh ethnic group was higher (11.5%) than that of the minority Ede ethnic group (8.6%). This tendency may be due to the Kinh ethic group possessing higher proficiency in the national language, and are thus, more likely to travel and had higher mobility as compared to other minority ethnic groups. This may reflect as increased risk of infectious disease exposure due to ethnic and socio-economical associated activity and behaviors. However, in the context of local socioeconomic development, rural poverty is still predominant, particularly in terms of access to medical care. These socioeconomic factors may further drive inter-city migrant workers to travel between larger cities and rural areas, in addition to low seroprevalence rate in the region, these factors may in turn lead to further Zika epidemic expansion during outbreaks.

Of the 718 samples that were negative for ZIKV IgM antibodies, 5 samples demonstrated high levels of ZIKV neutralizing antibodies with titers from 1: 320–1: 5120. Additionally, 3 samples were also positive for anti-ZIKV NS1 IgG, 2 of these 3 samples were confirmed by PRNTs with neutralizing antibody titers of 1:1280–1:5120. These results indicate local ZIKV transmission within the healthy community in the Central Highlands of Vietnam. Overall, 9/801 (1.1%) of the ZIKV confirmed case as positive for ZIKV had 5/200 (2.5%) cases collected in Cu Pong village, the same commune with the case of microcephaly cases that we reported in the previous study. In the vicinity at Chu Kbo and Pong Drang villages, only 2/119 (1.68%) and 2/202 (0.99%) positive cases were identified, respectively. This result indicates that the circulation of ZIKV in Central Vietnam is limited in the period of 2 years since the confirmation of nationwide ZIKV outbreak in Vietnam. In comparison with previous reports in Indochina, the ZIKV seroprevalence found in this study is at comparable levels with those of Cambodia [42] and Laos [43]. The low ZIKV seroprevalence, a proxy of protection against the disease in the community, however indicates that the community is at risk of subsequent ZIKV epidemic.

## Conclusion

This study confirms ZIKV infection in the Central Highlands of Vietnam and suggests that ZIKV has been present in the province since at least 2016. The prevalence of ZIKV-specific antibodies was 1.1% at the start of the study period, suggesting a limited outbreak within the area. Economic factors including migrant workers may play a role in introducing emerging pathogens such as ZIKV to rural areas. While DENV seroprevalence remains high in the region, the overall low ZIKV seroprevalence indicate limited Zika disease protection in the population. Further studies of seroprevalence in the general population and continuous surveillance are needed to better understand the extent of the outbreak in the general population and to define the potential risk of ZIKV transmission in the region.

## Data Availability

The datasets used and/or analyzed during the current study are available from the corresponding author on reasonable request.

## Abbreviations

DENV: Dengue virus
ELISA: Enzyme-linked immunosorbent assay
IgG: Immunoglobulin G
IgM: Immunoglobulin M
PRNT: Plaque-reduction neutralization test
ZIKV: Zika virus
